# Fighting the Monkeypox in Rwanda, An overview of current state and future recommendations

**DOI:** 10.1016/j.nmni.2024.101518

**Published:** 2024-10-30

**Authors:** Amos Kipkorir Langat, Maher Ali Rusho, Elysée Byiringiro, Godfred Yawson Scott, Amidu Alhassan, Vérité K. Cyubahiro, Christian Tague, Elie Kihanduka, Menelas Nkeshimana, Aymar Akilimali

**Affiliations:** Pan African University for Basic Sciences Technology and Innovation, Nairobi, Kenya; Department of Medical Biophysics, University of Toronto, Toronto, Canada; Faculty of Medicine, University of Rwanda, Kigali, Rwanda; Department of Research, Medical Research Circle (MedReC), Goma, Democratic Republic of the Congo; Department of Research, Medical Research Circle (MedReC), Goma, Democratic Republic of the Congo; Department of Medical Diagnostics, Kwame Nkrumah University of Science and Technology, Kumasi, Ghana; Department of Research, Medical Research Circle (MedReC), Goma, Democratic Republic of the Congo; College of Health and Allied Sciences, Department of Adult Health, University of Cape Coast, Cape Coast, Ghana; Faculty of Medicine, University of Rwanda, Kigali, Rwanda; Department of Research, Medical Research Circle (MedReC), Goma, Democratic Republic of the Congo; Department of Research, Medical Research Circle (MedReC), Goma, Democratic Republic of the Congo; Department of Health Workforce Development, Ministry of Health, Kigali, Rwanda; Department of Research, Medical Research Circle (MedReC), Goma, Democratic Republic of the Congo

**Keywords:** Emerging zoonotic disease, Health, Monkeypox, Public health, Rwanda

Monkeypox is a viral zoonosis caused by the monkeypox virus, part of the Orthopoxvirus genus, which also includes smallpox. The disease primarily occurs in Central and West Africa, but outbreaks have emerged in other regions, including Rwanda, due to increased global mobility and wildlife-human interactions. As an emerging zoonotic disease, it presents serious public health challenges, including transmission through contact with infected animals or humans, and possible secondary transmission within communities [1]. The monkeypox virus has two distinct genetic clades: the Central African and West African clades, with the former being more virulent and associated with higher mortality rates [2].

Rwanda’s health system faces unique challenges in handling monkeypox outbreaks, which have the potential to strain the system further due to limited resources. Understanding the frequency, distribution, and factors contributing to the spread of monkeypox in Rwanda is essential for preparedness. This study aims to provide a deeper understanding of the disease's dynamics in Rwanda, contributing to more effective strategies in prevention, early detection, and treatment.

Monkeypox was first identified in humans in 1970 in the Democratic Republic of Congo, but cases have since emerged in neighboring countries, including Rwanda. While data on outbreaks in Rwanda are limited, increased awareness and reporting suggest that cases have been identified sporadically. This section will focus on past outbreaks and how they have influenced current epidemiological trends within Rwanda [3].

Preliminary studies suggest that monkeypox cases in Rwanda are concentrated in rural areas where human-animal interactions are frequent. Demographically, the disease affects people of all ages, but there is a higher incidence among those involved in animal handling and farming. Understanding the geographical distribution will help in identifying high-risk areas for focused interventions [4].

Rwanda’s healthcare system faces challenges in addressing zoonotic diseases such as monkeypox, including limited diagnostic capacity, a lack of specialized healthcare personnel, and insufficient medical supplies. Additionally, the low vaccination rate for smallpox, which provides some cross-protection, leaves the population vulnerable [5].

Cultural beliefs and socioeconomic disparities play a role in the spread of monkeypox. Communities with traditional beliefs about disease causation may resist medical interventions, and rural poverty increases reliance on bushmeat, heightening the risk of animal-to-human transmission [2].

There is a need for improved data collection on monkeypox cases in Rwanda to better understand the epidemiology. Gaps in surveillance systems limit the ability to track the spread of the virus and evaluate the effectiveness of public health interventions [5].

## Future recommendations

o **Strengthening Surveillance and Early Detection**

To effectively manage future monkeypox outbreaks, Rwanda must strengthen its surveillance systems and early detection capabilities. This includes training healthcare workers in identifying symptoms and improving laboratory diagnostic capacity. Real-time reporting and data-sharing platforms will ensure a coordinated response [1].

o **Enhancing Community Engagement and Education**

Community-level education on the risks of monkeypox and its transmission should be enhanced. Engaging local leaders and using culturally relevant communication can improve understanding and compliance with prevention strategies [5].

o **Improving Vaccine Coverage and Treatment Access**

Although no specific vaccine for monkeypox exists, the smallpox vaccine offers some protection. Expanding access to vaccination in high-risk areas and ensuring sufficient medical treatment options are key to controlling outbreaks [1].

o **Research Priorities and Funding Opportunities**

More research is needed on the monkeypox virus, especially its epidemiology in non-endemic areas like Rwanda. Funding should prioritize studies on transmission dynamics, vaccine development, and the socio-cultural aspects influencing disease spread [2].

Monkeypox poses a significant threat to Rwanda’s public health infrastructure due to its zoonotic nature and potential for rapid spread in both rural and urban settings. Key findings from this study suggest the need for improved surveillance, enhanced community awareness, and the prioritization of vaccination campaigns to prevent future outbreaks.

The lessons learned from Rwanda’s handling of monkeypox can inform broader public health policies, particularly in resource-limited settings. Policymakers should consider integrating zoonotic disease preparedness into national health strategies, emphasizing capacity building and international collaboration to mitigate future risks.

## CRediT authorship contribution statement

**Amos Kipkorir Langat:** Data curation, Conceptualization. **Maher Ali Rusho:** Data curation, Conceptualization. **Elysée Byiringiro:** Formal analysis, Data curation, Conceptualization. **Godfred Yawson Scott:** Data curation, Conceptualization. **Amidu Alhassan:** Data curation, Conceptualization. **Vérité K. Cyubahiro:** Data curation, Conceptualization. **Christian Tague:** Data curation, Conceptualization. **Elie Kihanduka:** Data curation, Conceptualization. **Menelas Nkeshimana:** Data curation, Conceptualization. **Aymar Akilimali:** Writing – review & editing, Writing – original draft, Visualization, Validation, Supervision, Software, Resources, Project administration, Methodology, Investigation, Funding acquisition, Formal analysis, Data curation, Conceptualization.

## Data availability

Not applicable.

## Ethics approval and consent to participate

Not applicable.

## Provenance and peer review

Not commissioned, externally peer reviewed.

## Use of artificial intelligence tools

No artificial intelligence was used in generating the manuscript.

## Funding

The authors did not receive any financial support for this work. No funding has been received for the conduct of this study.

## Declaration of competing interest

The authors declare that there no conflict of interest.
